# Supplementary material for: Risk of disease and willingness to vaccinate in the United States: A population-based survey
Source: PLoS Med. 2020 Oct 15;17(10):e1003354. doi: 10.1371/journal.pmed.1003354 (PMC7561115; doi:10.1371/journal.pmed.1003354)
Supplement: S1 Text — (PDF) [file pmed.1003354.s002.pdf]

Appendix – Survey Questions

Dependent Variable:

On the following page, we will give you some information about a disease scenario in the United States. Please read the following material carefully. We will then ask you a few questions about this disease.

NOTE: SUBJECTS WERE RANDOMLY ASSIGNED TO ONE OF THE FOLLOWING EXPERIMENTAL CONDITIONS.

EXPERIMENTAL CONDITION 1
A new infectious disease has been discovered within the US. If you come into contact with an infected person, there is about a 25% chance that you will contract the disease. The disease can cause fever, diarrhea, vomiting, severe stomach pain, and neurological symptoms such as headaches and dizziness (but no risk of death). On average, people who contract the disease are sick for 1-3 days—too sick to work, go to school, care for others or leave the house. A highly protective vaccine (with minimal chances of side effects) is recommended and available to you locally at no cost.

EXPERIMENTAL CONDITION 2
A new infectious disease has been discovered within the US. If you come into contact with an infected person, there is about a 25% chance that you will contract the disease. The disease can cause fever, diarrhea, vomiting, severe stomach pain, and neurological symptoms such as headaches and dizziness (but no risk of death). On average, people who contract the disease are sick for 4-7 days—too sick to work, go to school, care for others or leave the house. A highly protective vaccine (with minimal chances of side effects) is recommended and available to you locally at no cost.

EXPERIMENTAL CONDITION 3
A new infectious disease has been discovered within the US. If you come into contact with an infected person, there is about a 25% chance that you will contract the disease. The disease can cause fever, diarrhea, vomiting, severe stomach pain, and neurological symptoms such as headaches and dizziness (but no risk of death). On average, people who contract the disease are sick for 8-14 days—too sick to work, go to school, care for others or leave the house. A highly protective vaccine (with minimal chances of side effects) is recommended and available to you locally at no cost.

EXPERIMENTAL CONDITION 4
A new infectious disease has been discovered within the US. If you come into contact with an infected person, there is about a 25% chance that you will contract the disease. The disease can cause fever, diarrhea, vomiting, severe stomach pain, and neurological symptoms such as headaches and dizziness (but no risk of death). On average, people who contract the disease are sick for 15+ days—too sick to work, go to school, care for others or leave the house. A highly

protective vaccine (with minimal chances of side effects) is recommended and available to you locally at no cost.

EXPERIMENTAL CONDITION 5
A new infectious disease has been discovered within the US. If you come into contact with an infected person, there is about a 25% chance that you will contract the disease. The disease can cause fever, diarrhea, vomiting, severe stomach pain, and neurological symptoms such as headaches and dizziness. The disease kills approximately 1 in 1000 people (0.1%) who contract it. A highly protective vaccine (with minimal chances of side effects) is recommended and available to you locally at no cost.

EXPERIMENTAL CONDITION 6
A new infectious disease has been discovered within the US. If you come into contact with an infected person, there is about a 25% chance that you will contract the disease. The disease can cause fever, diarrhea, vomiting, severe stomach pain, and neurological symptoms such as headaches and dizziness. The disease kills approximately 1 in 100 people (1%) who contract it. A highly protective vaccine (with minimal chances of side effects) is recommended and available to you locally at no cost.

EXPERIMENTAL CONDITION 7
A new infectious disease has been discovered within the US. If you come into contact with an infected person, there is about a 25% chance that you will contract the disease. The disease can cause fever, diarrhea, vomiting, severe stomach pain, and neurological symptoms such as headaches and dizziness. The disease kills approximately 1 in 10 people (10%) who contract it. A highly protective vaccine (with minimal chances of side effects) is recommended and available to you locally at no cost.

<u>Question: Local cases before vaccination</u>
There are lots of reasons why people do and don’t get vaccinated. Let’s focus on risk in terms of the number of reported cases of the disease in the places you live, work, or visit during a typical week. At which of the following infection levels would you get vaccinated?

1. I will vaccinate for this disease even if NO ONE is infected.
2. I will vaccinate for this disease if at least 1 person in the places I live, work, or visit is infected.
3. I will vaccinate for this disease if at least 10 people in the places I live, work, or visit are infected.
4. I will vaccinate for this disease if at least 100 people in the places I live, work, or visit are infected.
5. I will vaccinate for this disease if at least ___ people in the places I live, work, or visit are infected (please specify a number):____
6. I will NOT vaccinate for this disease. (You will be asked why in a follow up question).
7. I do not know.

Predictor Variables

Age: What is your age?

Gender: What is your gender?
- Male
- Female

Race: Which of the following describes your race? You can select as many as apply.
- White
- Black or African-American
- American Indian or Alaska Native
- Asian or Asian-American
- Native Hawaiian or Other Pacific Islander
- Some other race

Income: Last year, that is in 2017, what was your total family income from all sources, before taxes?
- Less than $10,000
- Between $10,000 and $19,999
- Between $20,000 and $29,999
- Between $30,000 and $39,999
- Between $40,000 and $49,999
- Between $50,000 and $59,999
- Between $60,000 and $69,999
- Between $70,000 and $79,999
- Between $80,000 and $89,999
- Between $90,000 and $99,999
- Between $100,000 and $149,999
- More than $150,000

Children: How many children have you ever had (either through birth, adoption, or guardianship)?
- None
- One
- Two
- Three
- Four
- Five
- Six
- Seven
- Eight or more
- I do not know.

Children Age: How old is your youngest child

- 1 year old or younger
- 2 years old
- 3 years old
- …
- ...
- .
- 22 years old or older

Hometown Size: Which of the following best represents where you live?

- Open country, farm, or rural place (population under 1,000).
- In a town or small city (1,000 – 50,000).
- In a medium-size city (50,000 – 250,000).
- In a large city (250,000 – 1 million).
- In a very large city (over 1 million).
- I do not know.

Commuter Status: During a typical week, do you commute to a different town or city for school, work, or personal business?

- Yes
- No

Commute Frequency (IF COMMUTER STATUS = YES): How many different towns/cities do you commute to in a typical week?

- 1
- 2
- 3
- 4
- 5
- 6
- 7
- 8
- 9
- 10 or more.

Size of City Commuted To (IF COMMUTER STATUS = YES): Think of the **first** town/city to which you commute in a typical week. Would you describe it as:

- Open country, farm, or rural place (population under 1,000).
- A town or small city (1,000 – 50,000).
- A medium-size city (50,000 – 250,000).
- A large city (250,000 – 1 million).
- A very large city (over 1 million).

Size of City Commuted To (IF COMMUTE FREQUECY > 1): Think of the **second** town/city to which you commute in a typical week. Would you describe it as:

- Open country, farm, or rural place (population under 1,000).
- A town or small city (1,000 – 50,000).
- A medium-size city (50,000 – 250,000).
- A large city (250,000 – 1 million).
- A very large city (over 1 million).

Size of City Commuted To (IF COMMUTE FREQUECY > 2): Think of the **third** town/city to which you commute in a typical week. Would you describe it as:

- Open country, farm, or rural place (population under 1,000).
- A town or small city (1,000 – 50,000).
- A medium-size city (50,000 – 250,000).
- A large city (250,000 – 1 million).
- A very large city (over 1 million).

NOTE: questions about fourth, fifth, sixth, seventh, eights, and ninth city are omitted here to preserve space.

Size of City Commuted To (IF COMMUTE FREQUECY > 9): Think of the **tenth** town/city to which you commute in a typical week. Would you describe it as:

- Open country, farm, or rural place (population under 1,000).
- A town or small city (1,000 – 50,000).
- A medium-size city (50,000 – 250,000).
- A large city (250,000 – 1 million).
- A very large city (over 1 million).

Education: What is the highest level of school you have completed or the highest degree you have received?

- Less than high school (Grades 1-8 or no formal education)
- High school incomplete (Grades 9-11 or Grade 12 with NO diploma)
- High school graduate (Grade 12 with diploma or GED certificate)
- Some college, no degree (includes some community college)
- Two year associate degree from a college or university
- Four year college or university degree/Bachelor’s degree (e.g., BS, BA, AB)
- Some postgraduate or professional schooling, no postgraduate degree (e.g. some graduate school)
- Postgraduate or professional degree, including master’s, doctorate, medical or law degree (e.g., MA, MS, PhD, MD, JD, graduate school)

Political Leaning: When it comes to politics, do you usually think of yourself as liberal, moderate, conservative, libertarian, or something else?

1. Very liberal
2. Liberal
3. Slightly liberal
4. Moderate / middle of the road
5. Slightly conservative
6. Conservative
7. Very conservative
8. I don't know / prefer not to answer
9. Libertarian
10. Other

Religion: Do you consider yourself Protestant, Roman Catholic, Jewish, or something else?

1. Protestant
2. Roman Catholic
3. Jewish
4. Mormon
5. Something else
6. I am not religious

Frequency of religious service attendance: Aside from weddings and funerals, how often do you attend religious services?

1. More than once a week
2. Once a week
3. Once or twice a month
4. A few times a year
5. Seldom
6. Never

Importance of Religion: How important is religion in your life?

1. Very important
2. Somewhat important
3. Not too important
4. Not at all important

Respondent Health: Would you say your own health, in general, is excellent, good, fair, or poor?

1. Excellent
2. Good
3. Fair
4. Poor
5. I don’t know.
