# Supplementary material for: Risk of disease and willingness to vaccinate in the United States: A population-based survey
Source: PLoS Med. 2020 Oct 15;17(10):e1003354. doi: 10.1371/journal.pmed.1003354 (PMC7561115; doi:10.1371/journal.pmed.1003354)
Supplement: S2 Text — (PDF) [file pmed.1003354.s003.pdf]

In order to test the robustness of our statistical analysis, we also performed analyses using a knowledge-driven approach. Given our initial motivations for our study, we developed models in which we investigate propensity to vaccinate as a function of: i) number of local cases (our surrogate for risk), ii) types of disease severity (morbidly and mortality), and iii) common socio-demographic predictors such as age, sex, education, income, religiosity, and ideology.

We developed four models. The first looked at only main effects. The second looked at interactions between our risk variable (number of local cases) and all others. The third took into account interactions between our scenario types (morbidity and mortality) and all others. Finally, we considered a model with all pairwise interactions between the main effects and both our risk variable (number of local cases, which we label as "nCases") and our scenario types (morbidity and mortality, which we label as "Scenario"). Our results are extremely similar across these models and to our original model: number of cases and scenario type are significant ($P < 0.01$), as well as age, sex, income, and political leaning. The one variable that our automatic approach (stepwise model selection) missed was related to commuting. The fact that our results are highly consistent across these different knowledge-based models (and our automatic model selection approach) suggest that our findings are robust across statistical approaches.

Below is a table that presents results from our knowledge-driven approach, including the main variables of interest and their interactions with the scenario type (Scenario) and number of local cases (nCases). The code for this analysis can be found at the end of the R file in the supplementary material.

	Degrees of freedom	X2	P(>|Chi|)
PolitcalLeaning	6	19.27	0.0037
Age	1	20.92	0.0000
Scenario	1	13.78	0.0002
Sex	1	10.72	0.0011
citySize	4	0.79	0.9397
Education	3	10.22	0.0168
Ethnicity	2	7.74	0.0209
Religion	3	4.52	0.2106
ReligionFrequency	5	2.24	0.8152
ReligionImportance	3	0.89	0.8274
RespondentHealth	3	1.31	0.7275
largestCommuteCity	5	6.16	0.2909
ChildStatus	2	1.48	0.4771
qIncome	3	14.89	0.0019
nCases	3	1209.77	0.0000
PolitcalLeaning:Scenario	6	4.84	0.5649
PolitcalLeaning:nCases	18	39.60	0.0024
age:Scenario	1	3.64	0.0566
age:nCases	3	26.28	0.0000
Scenario:nCases	3	6.32	0.0969
Scenario:Gender	1	0.01	0.9345
Gender:nCases	3	4.52	0.2101
Scenario:citySize	4	2.50	0.6447
citySize:nCases	12	14.10	0.2946
Scenario:Education	3	1.26	0.7395
Education:nCases	9	15.88	0.0694
Scenario:Ethnicity	2	1.05	0.5928
Ethnicity:nCases	6	4.61	0.5952
Scenario:Religion	3	1.40	0.7061
Religion:nCases	9	14.36	0.1102
Scenario:ReligionFrequency	5	4.69	0.4554
ReligionFrequency:nCases	15	12.41	0.6477
Scenario:ReligionImportance	3	4.38	0.2237
ReligionImportance:nCases	9	14.48	0.1063
Scenario:RespondentHealth	3	1.79	0.6161
RespondentHealth:nCases	9	18.82	0.0267
Scenario:largestCommuteCity	5	8.10	0.1510
largestCommuteCity:nCases	15	66.81	0.0000
Scenario:ChildStatus	2	2.28	0.3192
ChildStatus:nCases	6	8.64	0.1947
Scenario:qIncome	3	1.89	0.5961
qIncome:nCases	9	6.69	0.6696
