# Supplementary material for: Risk of disease and willingness to vaccinate in the United States: A population-based survey
Source: PLoS Med. 2020 Oct 15;17(10):e1003354. doi: 10.1371/journal.pmed.1003354 (PMC7561115; doi:10.1371/journal.pmed.1003354)
Supplement: Attachment — Submitted filename: Response to editors and reviewers.docx [file pmed.1003354.s006.docx]

Responses to requests from editors and comments from reviewers. Requests have been indented.

Requests from the editors:

You mention that you aim to "introduce" vaccine propensity in the present paper, but this has been mooted previously (PMID: 31461443). Please amend the presentation as appropriate.

The presentation has been amended. Several of the coauthors on this paper wrote the referenced paper, which was published around the same time as the submission of this paper. We have changed the language here to indicate that we are continuing to advance the concept of vaccine propensity and provided a reference to the 2019 paper.

Please adapt the title to better match journal style. We suggest: "Risk of disease and willingness to vaccinate in the United States: a population-based survey".

We thank the editors for the suggested title change. We have modified it slightly to highlight that we are interested in a dynamic rather than static picture. The title now reads: "Shifting risk of disease and willingness to vaccinate in the United States: a population-based survey".

Please expand the "methods and findings" subsection of your abstract, aiming to describe the methods and approaches used in additional detail in the early part of the subsection.

The “methods and findings” subsection of the abstract has been expanded to describe additional details, including the next two requests.

We ask you to quote aggregate demographic details for study participants in the abstract.

Done. (Mean age: 43.80; 47% male, 53% female, 38.5% with college degree, 24% non-White)

Please add a new final sentence to the "methods and findings" subsection of your abstract, which should summarize the study's main limitations.

Done.

Please begin the "Conclusions" subsection with "In this study, we found that ..." or similar.

The “Conclusions” subsection has been updated accordingly.

After your abstract, we ask you to add a new and accessible "author summary" section in non-identical prose. You may find it helpful to consult one or two recent research papers in PLOS Medicine to get a sense of the preferred style.

We thank the editors for suggesting to look at some recent research papers for guidance on style. We believe we have fulfilled this request.

Please streamline the "Introduction" section of your main text to reduce discussion of the Wakefield saga, for example.

The authors appreciate the suggestion to reduce discussion of the Wakefield saga. The

“Introduction” section of the main text has been revised.

Early in the "Methods" section, please state whether the study had a protocol or prespecified analysis plan, and if so attach the relevant document(s) as a supplementary file. Please highlight analyses that were not prespecified.

We have added statements at the beginning of the “Methods” section that our study did not have a protocol or prespecified plan with respect to our goal of empirically advancing the concept of vaccine propensity.

Noting the "table sample vs census" in the supplementary material, we suggest that you include a table presenting the characteristics of the study participants, preferably including absolute numbers of participants, early in the "Results" section. Please rephrase "White Alone".

The table “sample vs census” has been moved to results, “white alone” has been changed, and absolute numbers have been provided.

Please restructure the early part of the "Discussion" section of your main text: the first paragraph should consist mainly of a summary of the paper's findings.

The “Discussion” section now begins with a paragraph that concisely summarizes the paper’s findings.

Also, we ask you add a discrete discussion paragraph on study limitations.

A discrete discussion paragraph about study limitation has been added towards the end of the discussion section.

Please substitute "sex" for "gender", where appropriate, throughout your paper.

All instances of “gender” in the manuscript have been replaced with “sex” as appropriate.

Please avoid using italics for emphasis.

Italics for emphasis have been removed.

In the reference list, please abbreviate journal names consistently (e.g., "PLoS Med." for reference 4).

Done.

Noting reference 11, please ensure that all references have full access information.

Done.

Noting "S3 file", we suggest removing the IP addresses and any other information which could be used to identify study participants. Please let me know if it would be helpful to discuss this further.

The IP address column is now empty.

Please adapt your attached SRQR checklist so that individual items are referred to by section (e.g., "Methods") and paragraph number rather than by page or line numbers, as the latter generally change in the event of publication.

Done.

Comments from the reviewers:

*** Reviewer #1: 
The authors report the results from a single online experimental study examining willingness to vaccinate for a novel, hypothetical disease. The design allows in principle for exploration of both variations across demographics and political attitudes and variation over severity of disease (through a between-subjects manipulation of the description in to 7 groups, 3 with differing degrees of mortality and 4 with varying duration of non-life threatening illness) and likelihood of exposure (through a response question that incorporated information about local cases). The authors frame their inquiry as introducing a new concept ("vaccine propensity"). While there is some value to further explorations of correlates to vaccination intention / attitudes, I have both conceptual concerns about what is being measured here and substantial uncertainty about the significance of the findings. 
To start, I don't really see how "vaccine propensity" is a novel construct. Many authors (including some cited in this paper) have explored likelihood of vaccination as various individual and situational factors are changed (e.g., in discrete choice experimental structures). The present authors take a novel approach to measurement, but it remains unclear how readers should think about what is being measured here and how it will relate to real world behaviors.

Reviewer #1 is correct that “vaccine propensity” has been explored in terms of the likelihood of vaccination as other factors are changed, including papers that we cite. Furthermore, shortly after the submission of this paper a publication that explicitly uses the phrase “vaccine propensity” was released and was in fact written by some of the authors in this paper. Reflecting on the reviewer’s comments, we have described the present work as empirically advancing the concept of vaccine propensity. As the reviewer correctly identifies, what we see as novel is the measurement (at the individual level) of a population-level phenomenon in which, absent institutional changes, disease outbreaks lead to increases in vaccination rates. This increase in vaccination rates is presumably due to increase in risk perception and our aim was to better understand how this risk perception is linked to number of local cases, holding fixed several other considerations. (This last point is related to Reviewer #1’s second comment, which we discuss below.) We have revised the “Introduction” and “Discussion” sections to reflect these considerations.

With respect to the reviewer’s concern about “how it will relate to real world behaviors” one example we have in mind is outbreaks of measles in recent years and how epidemiologists can better understand and predict them. Do to that we need better models that account for the feedback between human behaviors and disease spread. One way to link these is through case counts, as illustrated by the fact that case counts are the predominant source of information reported in media. Several of the authors of this paper are working on developing such models and are currently working on a manuscript to this end. The authors, however, feel it would take the current paper too far astray to discuss the real world relevance beyond what is currently said in the final paragraph of the “Discussion” section. We thus ask the reviewer to forgive us for not taking up this point further.

Second, I have a conceptual problem with the design of this study. The authors "we asked each respondent how many people in his/her local community would have to get infected with this disease for him/her to get vaccinated" and then provided response options that listed different numbers of local cases. The structure of the question not only assumes that the proportion of people affected in their local community is a variable that ordinarily affects people's decisions, it implies directly that it should be. Thus, I see strong potential for a demand effect, in which respondents use the local case information because they are told to do so. There is also a huge potential for bias based on the set of response options provided (consistent with the literature on option set biases in survey design.) In other words, I am not confident that changes in the response option set would not change the results. Thus, what is measured is which respondents react to a particular set of local
case count options, and the graphics are showing the increase in cumulative willingness to vaccinate as we add each group of responses.

The reviewer is correct in identifying that our study assumes that local case counts is a variable that ordinarily affect’s people’s decision. This assumption is motivated by evidence that disease outbreaks increase risk perception and vaccination rates, independently of institutional changes (we summarize some relevant literature in the introduction). Moreover, case counts are a predominant piece of information presented in the media because that is presumably what their audiences care about. Nevertheless, we understand the reviewer’s concern and we acknowledge this as a potential limitation of our study. We have described this limitation in the discussion section (see paragraph on the limitations of our study).

The authors collapse over their 7 scenarios into either mortality or morbidity, thereby ignoring the variance in degree of either outcome. This appears to be because the degree did not affect results (per the second paragraph of the discussion but never discussed or demonstrated in the results). Nonetheless that should be shown especially since it is interesting to consider how long duration morbidity compares to rare mortality.

The reviewer is correct. We mention in the discussion that there were no significant differences when varying the severity of morbidity or mortality. However, we respectfully disagree with the view that non-significant results should be presented. By virtue of being “non-significant,” we cannot distinguish the effect of these covariates from zero, thus it seems misguided to belabor such results other than to mention that they were not significant. If the editors feel differently, we will happily include discussion of nonsignificant results. Either way, we have added a brief discussion of this in our study limitations.

In terms of results, I'm left with very unclear takeaways. Mortality evokes slightly stronger reactions than morbidity (surprising no one, since a disease that kills is clearly more severe than one that does not). However we have no sense whether we should see this difference as larger or smaller than appropriate. More local cases evokes stronger vaccination interest, which is expected because local prevalence increases chances of exposure and because it serves as an indirect measure of disease scope. (I suspect many people are using this value as a measure of infectiousness, in replacement of the provided 25% rate.) The slope of this effect, however, is dependent on the hypothetical scenario context, the parameters used, and the option set presented, which limits how much readers can generalize from it to the real world. The age gradient suggests that older adults are just generally more likely to want to vaccinate regardless whereas younger adults are more situation
dependent. This is a mildly interesting finding, consistent with the literature on differences in processing styles and decision making over the life course.

We thank the reviewer for providing the opportunity to clarify this. We have revised the “Discussion” section to clarify the main takeaways of this study, as well as the limitations of our study. Specifically, we have added a concise summary paragraph at the beginning of the “Discussion” section (as per requested by the editors) and we have included a paragraph towards the end that outlines the limitations. We also hope that the addition of the Author Summary helps in this respect.

I agree that the political ideology information probably the most interesting part of these findings, and a paper that focused primarily in this domain might be of more interest to me.

We agree with Reviewer #1 that this finding is perhaps the most interesting. It may interest Reviewer #1 to know that the authors here have explored the connection to ideology elsewhere (Baumgaertner, 2018) and so to some extent it was not surprising to see a relationship between vaccine propensity and ideology. That said, our interest in this paper was not just ideology, but included a wider swath of sociodemographic information. Moreover, we hope that Reviewer #1 can appreciate that we are balancing their comment against another reviewer’s request to increase breadth. We have thus decided to maintain the breadth of this paper as is and will take Reviewer #1’s comment as a prompt for future avenues of research.

Minor points:

The authors change their Y axis scaling from graphic to graphic, thereby distorting readers' perceptions of the magnitude of the changes shown. This is poor communication practice in general, but it is particularly pernicious in this context where the effect sizes are (a) only involving changes of roughly .1 and (b) variable across analyses.

We have now rescaled the axes on figure 2-4 to be the same; we have done likewise for figures 5-7. We have updated the figure descriptions to make clear the two scales we have selected.

I'm also concerned by the authors' reframing of the local case information as "risk." While there is a relationship between local cases and likelihood of exposure, "risk" is a broader construct that this. The authors should remain concrete in their descriptions: this is a manipulation of local cases.

The authors agree and have made revisions where appropriate to reflect the fact that where this paper is concerned “risk” is understood in terms of local cases. Specifically, additional care has been taken in the Introduction (para. 8), Results (para. 1), and Discussion (paras. 1 and 3) sections.

There is little to no discussion of limitations, which is a significant omission given the many challenges in trying to generalize these findings beyond this particular survey.

Limitations are now discussed in the Discussion section (para. 10).

*** Reviewer #2: 

This paper investigates population willingness to consider vaccination using a set of vignette questions administered to an online sample of adults in the US, and variations across demographic subgroups.  The paper is well written and well-reasoned. In this review, I focus on methodological aspects of the study and leave substantive concerns to vaccination experts. Specific questions for the authors include the following:
*    How many survey respondents (and what %) failed the three quality control checks?

58.5 percent of people who clicked on our survey (3007 of 5141 individuals) passed all quality control checks. We have added this information in the Data Collection section.

*    What was the survey response rate, and how was it calculated? Most journals now require that the specific formula be reported, such as those provided by the American Association for Public Opinion Research.

Our data provider (Survey Sampling International, or “SSI”) generates nationally SSI does not report the response rate for its surveys. Instead, the questionnaire automatically closes for respondents in particular socio-demographic subgroups once this subgroup reaches a pre-determined quota. The representative respondents are pooled by sending survey links to the members of a U.S-wide online panel. We have added this information in the first paragraph of the “Data Collection and Sample Characteristics” section.

*    Was the final sample weighted and were those weights used for all or some of the analyses reported?

Given that our survey generated a nationally representative sample of the U.S. adult population, observations were not weighted.

*    How confident are the authors that their sample will respond to hypothetical threat questions in a manner similar to how they would respond to a real threat? Many survey research experts frown on hypothetical questions because they have been found not to be predictive of subsequent behavior. This is, at a minimum, a limitation that should be acknowledged if not more aggressively addressed.

We agree with the reviewer that this is a limitation of our study and have included this in our “Discussion” section.

*    Actually, the paper does not include a discussion of study limitations. This needs to be corrected.

We agree and apologize for this omission. A discussion of study limitations is now included in the “Discussion” section.

*    The number of respondents answering that they did not know what the likelihood was that they would vaccinate (n=395) seems sufficiently large that it might be informative to examine the demographic variables associated with that uncertainty as well. This could probably easily be accomplished with the data at hand.

We appreciate the reviewer’s suggestion. We hope that the reviewer will understand that we are trying to balance the breadth of our paper by another reviewer’s request to focus more narrowly. In light of all requests by reviewers and editors, we have decided to maintain the breadth of our paper as is. That said, the data will be made available and we encourage both Reviewer #1 and others to explore these sorts of questions.

*    Sample sizes should be provided for the models shown in Tables 2 & 3.

Done. Note that as a consequence of moving the “Sample vs Census” table to the results section, Tables 2 & 3 in the original submission are now Tables 3 & 4, respectively.

*    The final supporting table, that compares the sample with benchmark data, does not include education, and this seems to be a serious omission.

We thank the reviewer for pointing this out. It was in fact not an omission because the table (in its original form) only compared our sample with variables for which we ensured nationally representativeness. However, we have decided to include education in our table and made clear that while Survey Sample International was not able to guarantee a match, the numbers happen to be relatively close. In response to other feedback, we have moved this table into the results section and added language to clarify how education was related to our survey design.

*    Relatedly, another important limitation, not acknowledged, is that the study sample purports to be nationally representative, but provides no information about the sample frame for the online survey that was conducted. Given that many Americans do not access the internet, the burden of proof is on investigators to make a convincing case that the sample is indeed nationally representative, as the paper's title implies.

The reviewer is correct that given the online nature of our survey our sample is not a perfect representation of the US population. We have added this to our discussion of study limitation. That said, in line with existing survey research we have ensured that our respondent pool matches known population parameters on five important dimensions (age, sex, income, race, and region). We have moved the table (“Sample Characteristics”) to the main manuscript which demonstrates that this goal was achieved.

*** Reviewer #3: 

I confine my remarks to statistical aspects of this paper. The general approach is fine but I have several issues to resolve before I can recommend publication.

Line 199 -- How were ordinal values treated?  (they are tricky)

Ordinal values were treated as ordinal values. In other words, the k-classes are ordered and the logit link function is used giving log(P(Y<=k) – log(P(Y>k)). If further details for R’s treatment of ordinal factors is of interest, we recommend the book “Modern Applied Statistics with S-Plus” by Venables and Ripley.

Line 205 - How much data was "missing"? Just deleting it is not usually good, unless there is very little missing

Of the 5140 surveys we collected, 66 were discarded due to missing information.

Lines 217-219  Don't further clump income this way. This increases both type I and type II error.

If we understand what the reviewer is saying here, we agree with them but are not sure what action to take. Aggregating responses changes the variance structure of the data. However, categorical response for things like income are already a substitute for a continuous variable; so getting at some sort of "true" relationship with income level isn't possible. We are therefore already limited with what we can infer from the data. By aggregating and changing the variance structure of the income classification (say into 10k increments), we are changing the hypothesis being tested. In other words, the variance structure is no longer appropriate for testing hypotheses about the 10-classes of income, but it IS appropriate for testing hypotheses about the new 4-classes of income. Also, it is important to recognize that income is a continuous variable and not one where people are forced to make subjective decisions about which category something should be in; i.e., if we had originally presented individuals with the 4 choices of income level, their responses should (in theory) exactly match the aggregated version of the more finely divided data.

Lines 225-227  Can it be right to treat others as having graduate education? Maybe it is, but this needs some explanation.

We thank the reviewer for the opportunity to avoid a potential miscommunication. The original phrase was, “finally, anyone else” which we did not intend to imply as “other” but rather we meant to say “those who did complete a graduate/professional degree”. We have corrected this language.

Line 236-238  Do not use stepwise selection. All the results will be wrong. P values are too low, standard errors are too small, parameter estimates are biased away from 0.... See e.g. Harrell *Regression Modeling Strategies*. Instead, substantive knowledge should be used to build models. If the authors insist on an automatic method, LASSO is much better than stepwise (but not that good).

We appreciate that there is an ongoing debate as to whether model selection gets you closer to a true model. However, we choose to abstain from this debate that has gone on for decades. We also acknowledge that the use of model selection is a routine process. There are many different ways of choosing models; LASSO, ridge, elastic-net, divergence statistics, other penalized likelihood, etc. We are of the mind that model selection is a valuable technique for large datasets. We thus respectfully disagree with the reviewer here and appreciate that they acknowledged that our “general approach is fine” at the start of their review.

Table 2  There is no real R^2 for logistic regression. There are various alternative measures, none of which are perfect.  Which was used?

The reviewer is correct. This is a pseudo-R^2. We use the specific variant found in

Efron, B. (1978). Regression and ANOVA with zero-one data: Measures of residual variation. Journal of the American Statistical Association, 73, 113-121.

We have clarified that this is pseud-R^2 and added the reference in the paper. Note that this is now Table 3 (previously Table 2).
