# Supplementary material for: Risk of disease and willingness to vaccinate in the United States: A population-based survey
Source: PLoS Med. 2020 Oct 15;17(10):e1003354. doi: 10.1371/journal.pmed.1003354 (PMC7561115; doi:10.1371/journal.pmed.1003354)
Supplement: Attachment — Submitted filename: Response to editors and reviewers.docx [file pmed.1003354.s007.docx]

Feedback from editors and reviewers have been copied in full. Responses follow each point of feedback, beginning with an indentation.

-----------------------------------------------------------
Requests from the editors:

1.Title: Thank you for revising your title.  However as your survey was cross-sectional and none of your results speak to any time course of changing attitudes, we feel that the use of the word “shifting” is not congruent with the study. Again, we suggest: "Risk of disease and willingness to vaccinate in the United States: a population-based survey".

We have revised the title as suggested.

2.Abstract: Methods and Findings: Please present the study methods before the study findings.  In the limitations sentence, please clarify what is meant: “...it does not consider how other considerations interact with local case counts in people's vaccine decision making…” as it is not clear what is meant by ‘other considerations’

The methods are now presented before the study findings. We have added an example of what is meant by “other considerations” in parentheses. It now reads, “…other factors (such as social influence)…” We have likewise updated the second last paragraph of the Discussion section where we describe limitations.

3.Author Summary: Thank you for including an author summary.  Please structure the author summary using bullet points for separate ideas within the three sections, rather than writing in paragraph format. Please see our author guidelines for more information: [https://urldefense.com/v3/__https://journals.plos.org/plosmedicine/s/revising-your-manuscript*loc-author-summary__;Iw!!JYXjzlvb!yTzgP2rs7-1QuSPuqE8XRnDzKa2axSi7RpzCfyM2ASt1ljmFaFV9icSLfeb5pKw$](https://urldefense.com/v3/__https:/journals.plos.org/plosmedicine/s/revising-your-manuscript*loc-author-summary__;Iw!!JYXjzlvb!yTzgP2rs7-1QuSPuqE8XRnDzKa2axSi7RpzCfyM2ASt1ljmFaFV9icSLfeb5pKw$) 

The Author Summary is now structured using bullet points.

4.Methods: Line 118-119: It is not satisfactory to state that: “With respect representativeness, we outsourced the distribution of our survey to Survey Sampling International which utilizes proprietary methods.”- please provide details or a reference to the methods relating to the survey administration including response rates, and also see the comments of reviewer 2.  If response rates are not available due to the limitations of SSI, please address the concerns of reviewer 2 and devote substantial space to describing how this impacts the interpretation of the study’s findings as a paragraph of the limitations section of the Discussion.

We have substantially revised this first paragraph of the subsection “Data Collection and Sample Characteristics”. It now provides more details (including completion rates) that we were able to obtain about SSI’s methodology when collecting our sample. Relatedly, we agree with Reviewer 1’s suggestion to replace all instances of “nationally representative” to “demographically diverse”. This language better reflects the nature of our sample and our methods, and it is also consistent with other PLOS publications we consulted that also used SSI (links below). In addition, we believe this will also help address the concerns of Reviewer 2 regarding “representativeness” and statistical weighting.

If the editors or reviewers are interested, the PLOS publications we consulted can be found at the links below.

<https://urldefense.com/v3/__https://journals.plos.org/plosone/article?id=10.1371*journal.pone.0231768__;Lw!!JYXjzlvb!1grwesVCrvReKIq73yZp9mahYC28DVF8oVmZu7nQi1Lu2iqNwbXDjdV5IH6pyNo$>

<https://urldefense.com/v3/__https://journals.plos.org/plosone/article?id=10.1371*journal.pone.0097506__;Lw!!JYXjzlvb!1grwesVCrvReKIq73yZp9mahYC28DVF8oVmZu7nQi1Lu2iqNwbXDjdV5chBf720$>

<https://urldefense.com/v3/__https://journals.plos.org/plosone/article?id=10.1371*journal.pone.0213668__;Lw!!JYXjzlvb!1grwesVCrvReKIq73yZp9mahYC28DVF8oVmZu7nQi1Lu2iqNwbXDjdV5xD2dR-M$>

<https://urldefense.com/v3/__https://journals.plos.org/plosone/article?id=10.1371*journal.pone.0133779__;Lw!!JYXjzlvb!1grwesVCrvReKIq73yZp9mahYC28DVF8oVmZu7nQi1Lu2iqNwbXDjdV5rXQJGRo$>

<https://urldefense.com/v3/__https://journals.plos.org/plosone/article?id=10.1371*journal.pone.0118489__;Lw!!JYXjzlvb!1grwesVCrvReKIq73yZp9mahYC28DVF8oVmZu7nQi1Lu2iqNwbXDjdV55G-GZWo$>

5.Discussion: Line 333-336 (and similarly at line 369): Please remove the suggestion of a causal relationship from this sentence, and throughout, we suggest the following change: “Thus, the goals of this paper were: to empirically advance the concept of vaccine propensity to enrich our understanding of complacency in vaccine hesitancy, and to study the sociodemographic factors that are associated with vaccine propensity."

We very much appreciate this suggestion as it was not our intention to imply a causal relationship. We have replaced “affect” with “are associated with” as recommended.

6.References: Please use the "Vancouver" style for reference formatting, and see our website for other reference guidelines [https://urldefense.com/v3/__https://journals.plos.org/plosmedicine/s/submission-guidelines*loc-references__;Iw!!JYXjzlvb!yTzgP2rs7-1QuSPuqE8XRnDzKa2axSi7RpzCfyM2ASt1ljmFaFV9icSLFp4_UrM$](https://urldefense.com/v3/__https:/journals.plos.org/plosmedicine/s/submission-guidelines*loc-references__;Iw!!JYXjzlvb!yTzgP2rs7-1QuSPuqE8XRnDzKa2axSi7RpzCfyM2ASt1ljmFaFV9icSLFp4_UrM$)  
(for example, journal title is inconsistent in #4 and #18).

We have changed #4 to match the ISO abbreviation (PLoS Med.) that is found in NCBI Databases.

Regarding the “Vancouver” style for reference formatting, we believe this is already the default in the PLoS LaTeX template we are using (the bibliography style package is called plos2015). We apologize for any confusion on our part and welcome suggestions for how to make further corrections.

7. Table 1: The “Income” measure requires units.

The line in the table now reads “Income (in USD)”.

8. Table 2: Income, hometown size, commute city size need units associated with the values presented.

The relevant lines now read: “Income (in USD)”, “Hometown population size”, and “Largest city population size commuted to”.

9. Table 3: Please provide the abbreviation for “df” in the legend.

We have now spelled out the abbreviation: Degrees of freedom

10. Table 4: Please provide the abbreviation for “SE” in the legend.

We have now spelled out the abbreviation: Standard Error

11. Figure 2: Please see reviewer 1’s comments regarding the axes of graphs, and please begin the y-axis at zero (in this case, the axis should display from 0 - 1.0).  Please explain why the points are offset from the “1” “10” “100” lines, etc.  Please describe the purpose of the dotted line connecting the points in the legend.

This figure now has the y-axis go from zero to one.

The small offset is from the Jitter operation in R. These small offsets and the dotted lines are for visual aid. We have added the following explanation in the caption: “Slight offset in points and dotted lines are for visual aid.”

12. Figure 3: Please see reviewer 1’s comments regarding the graph axes  (in this case, the axis should display from 0 - 1.0).  Please explain why the points are offset from the “1” “10” “100” lines, etc. In the legend, please define the whiskers extending from the points.

This figure now has the y-axis go from zero to one.

We have added the following explanation: The points are offset from 0, 1, 10, and 100 as a visual aid. Whiskers from the points are the 95% confidence intervals.

13. Figure 4: Please see reviewer 1’s comments regarding the graph axes  (in this case, the axis should display from 0 - 1.0).  Please explain the nature of the solid lines in the legend- are these trendlines?  Please explain the shaded regions in the figure legends.  As these are difficult to see, we suggest using different colors.

This figure now has the y-axis go from zero to one.

We darkened the shaded regions so that they are now better seen.

We added the following explanation: The solid lines are the trend lines and the shaded area is the 95% prediction interval.

14. Figure 5: Please see reviewer 1’s comments regarding the graph axes  (in this case, the axis should display from 0 - 1.0).  Please explain the nature of the solid lines in the legend- are these trendlines?  Please explain the shaded regions in the figure legends.  As these are difficult to see, we suggest using different colors.

This figure now has the y-axis go from zero to one.

We darkened the shaded regions so that they are now better seen.

We added the following explanation: The solid lines are the trend lines and the shaded area is the 95% prediction interval.

15. Figure 6: Please see reviewer 1’s comments regarding the graph axes (in this case, the axis should display from 0 - 1.0).  Please explain the nature of the solid lines in the legend- are these trendlines?  Please explain the shaded regions in the figure legends.  As these are difficult to see, we suggest using different colors.

This figure now has the y-axis go from zero to one.

We darkened the shaded regions so that they are now better seen.

We added the following explanation: The solid lines are the trend lines and the shaded area is the 95% prediction interval.

16. Figure 7: Please see reviewer 1’s comments regarding the graph axes (in this case, the axis should display from 0 - 1.0).  In the legend, please define the whiskers extending from the points.

This figure now has the y-axis go from zero to one.

We have added the following explanation: Whiskers from the points are the 95% confidence intervals.

17. Supporting information file S3: It appears that you have included data that may breach participant confidentiality. Please remove potentially identifying information from the file (e.g. IP address, county and zip codes, latitudes and longitudes, etc.)

We sincerely apologize for this. Apparently we mistakenly uploaded the same file from our first submission. The new file S3 no longer includes any identifying information. We have removed IP addresses, county and zip codes, and latitudes and longitudes.


Comments from the reviewers:

Reviewer #1: This manuscript is a revision of a paper I reviewed previously. The authors have addressed a number of the concerns raised previously about the framing of their work and its implications. I still find the paper less focused than I would prefer, but this is an editorial choice. 

One issue that I believe was insufficiently addressed, however, is axis scaling in their figures. The authors state: "We have now rescaled the axes on figure 2-4 to be the same; we have done likewise for figures 5-7. We have updated the figure descriptions to make clear the two scales we have selected.." Figures 2-4 are scaled from roughly .3 to 1.0 despite the fact that it is reporting a propensity. There is no logical reason to use this scale (vs. the full 0-1.0 range that a propensity can vary over) except to magnify slope, which is amplifying readers' perceptions of the effect. Figures 5-7 are scaled from roughly 0.6 to 0.95. Same issue, same effect, same problem, except even more so. Worse, Figure 4 is of the same type as Figures 5-7, but it is on a different scale. Thus, the slope represented in Figure 5 for morbidity (a range of roughly 0.15 over the age distribution) looks significantly steeper than the slope in Figure 4 for 1 local cases (a range of roughly
0.12-0.15) despite the fact they show numerically very similar levels of variation. This is clearly distorting perceptions of results. Let me be clearer than I was previously. In reporting propensity, I find it hard to justify any Y axis scaling other than 0-1.0.

We acknoweldge Reviewer 1’s concern. We have changed all the relevant plot Y-axes to be on a 0-1.0 scale.

I also have a few minor but important issues that need resolution:

1) Kudos to the other reviewer for flagging an issue I should have caught the last time around: the presentation of this sample from SSI as "nationally representative". I acknowledge that the authors have now put a note in their limitations on this issue. However, speaking as a frequent user of online samples, I _never_ describe an SSI sample or any other online sample (except maybe GfK) as "representative" of anything. It is demographically diverse in ways that mirror US Census demographics, yes, and the authors took specific steps to achieve this (with clear success). But it is not "representative" not only because of limitations on access to the internet but because it is an opt-in panel: SSI recruits people who want to take surveys, which is not everyone, and this is a characteristic that plausibly might relate to survey responses on this type of scenario. Thus, this is a minor but to me important point: I ask the authors to remove all descriptions of their sample as
"nationally representative" and frame it instead as "demographically diverse" or similar.

We agree with Reviewer 1 and have framed the sample instead as “demographically diverse”. Thank you for this helpful suggestion, which we found is also consistent with how other PLOS publications have framed their samples that were collected with the help of SSI (links provided above in our response to the editors).

2) It's not just that survey providers like SSI don't report response rates, it's that their methodology doesn't align to the concept. I believe SSI is no longer sending links to participants (which participants would either reply to or not) but instead basically encourages panel members to sign on to their account and then surveys are dynamically assigned to them moment by moment. This approach (or any like it) does not have a true denominator for use in determining a response rate. I would reword this to clarify more details of the sampling process in place at that time.

We were able to obtain a more detailed explanation for how SSI collected our sample. We have substantially revised the first paragraph of the subsection “Data Collection and Sample Characteristics” to include this information. (In the marked up version, the old paragraph is in red, the new paragraph is in blue.)

3) What does matter for online survey quality, however, is completion. The authors report that over 40% of their respondents failed quality control checks and were omitted from analyses. That is a huge number, and hence readers deserve a bit more detail on the character of those checks so as to judge the implications.  In addition, it represents one more way in which the analytical sample is not representative (per comment above), since a representative sample would include at least some people who fail those attention checks.

These are good points and we agree that such details should be provided. As Reviewer 2 also suggested, we contacted SSI do obtain additional information. We have included additional details in the revised paragraph under “Data Collection and Sample Characteristics”. We have also acknowledged in the limitations paragraph (second last in the Discussion section) that our study is unable to capture those who did not complete the survey or failed the attention checks.


Reviewer #2: Thank you for continuing to work on this paper, which has been considerably improved. There are several methodological questions regarding data collection, though, that remain inadequately addressed. These problems largely stem from the sample employed for the survey, how it was selected, what proportion of sampled respondents participated, and how the sample was weighted for analysis. Below, these concerns are summarized.

*	Inability to report response rates suggests a poor methodology that more than anything seriously challenges any claims that the sample is representative of anything. I would encourage the authors to push back on the survey group they worked with to obtain and report a convincing rationale for this complete lack of transparency. My guess is that the only potential reason for being unable to provide a response rate is the use of a very poor convenience sampling methodology. My personal opinion is also that failure to provide response rate information is grounds for rejection. Appreciate that the sample's demographics have been compared with Census data. However, one key measure is not included in those comparisons: the percent of the population that does and does not have internet access. There is no question that the sample will be unrepresentative of this characteristic.

We took Reviewer 2’s suggestion to push back on the survey group (SSI) and we were able to obtain a number of details about the sampling methodology. (Thank you for encouraging us to do this.) We have included the details we were able to obtain in a substantially revised paragraph, the first one under “Data Collection and Sample Characteristics”.

With respect to representativeness, we apologize for confusion on our part. Reviewer 2 is correct that there are various ways in which we simply cannot be representative given the nature of our sample, including the part of the population that does not have internet access, as well as (as Reviewer 1 also pointed out) those who did not complete our survey or failed our attention checks. We have thus adopted the suggestion to frame our sample as “demographically diverse” and acknowledged ways our sample is not representative in our limitations paragraph (second last in the Discussion section).

*	Providing a discussion of limitations is appreciated and improves the paper. Lack of a response rate, which leaves the authors unable to even begin to address nonresponse bias concerns, is missing from this discussion, however.

We agree and believe the revisions we mentioned above help address this.

*	Failure to properly weight the sample also detracts from claims that findings are representative. Even the most carefully designed nationwide probability surveys include statistical weights. That a nonprobability sample does not need to use weights because it is already "representative" is laughable.

Upon reflecting on this feedback, and given the additional feedback from Reviewer 1, we came to the decision that it is better to interpret our sample as demographically diverse rather than push for the claim that it is representative. After all, our sample does not include individuals that might be expected to be included in a properly representative sample, such as those without internet, and so it would be impossible to “properly weight” the sample accordingly. If Reviewer 2 has something specific in mind for how we might address this issue, we welcome suggestions. However, we think Reviewer 2 will find it acceptable that we opted to walk back from claims of representativeness. And as mentioned above, we have revised our discussion of the sample and acknowledged the relevant limitations of interpreting our results.

*	Another relevant topic that remains unaddressed is the question of the frame from which the sample for the survey was selected. Exactly how do individuals get recruited into the SSI sample frame? It is almost certainly not a random process and the reader is left to guess or search the internet for relevant information. I did look at the SSI (now Dynata) web site and was unable to locate anything helpful.

We again thank Reviewer 2 for suggesting that we push back on SSI. We were able to obtain some details from SSI and we have added these in the first paragraph under “Data Collection and Sample Characteristics”.

Reviewer #3: I am checking "Proceed without recommendation" because I am a little confused.

The authors have addressed most of my concerns. Remaining issues:

I wrote: Lines 217-219 Don't further clump income this way. This increases both type I and type II error.

The authors responded: If we understand what the reviewer is saying here, we agree with them but are not sure what action to take. Aggregating responses changes the variance structure of the data. However, categorical response for things like income are already a substitute for a continuous variable; so getting at some sort of "true" relationship with income level isn't possible. We are therefore already limited with what we can infer from the data. By aggregating and changing the variance structure of the income classification (say into 10k increments), we are changing the hypothesis being tested. In other words, the variance structure is no longer appropriate for testing
hypotheses about the 10-classes of income, but it IS appropriate for testing hypotheses about  the new 4-classes of income. Also, it is important to recognize that income is a continuous
variable and not one where people are forced to make subjective decisions about which category something should be in; i.e., if we had originally presented individuals with the 4
choices of income level, their responses should (in theory) exactly match the aggregated version of the more finely divided data.

My further response.  Certainly changing the variable changes the variance structure. But .... I'm confused as to why that means the authors have to use income as a 4 category variable rather than a continuous one or a 10 category one. How was income recorded in the original data? Was it continuous (that would be unusual) or was it 10 category (in which case, I still don't see why they changed it). I feel like I am missing something here. Clearly the authors are proficient at statistics, but I don't quite understand their reasoning.

We apologize for not being sufficiently clear about this. We did not “have to” use income as a 4 category variable. We suppose we could have instead used a 5, 6, …, 10 category variable (the original data was 10). For our purposes and interest, we don’t see any significant advantages or disadvantages of doing it one way or the other. (For what it’s worth, both the 10 category and 4 category versions had just about uniform distributions over the categories.) Our thinking was, in the absence of substantial reasons for one over another, we opted for a 4 category variable because it helped decrease the number of degrees of freedom.

-----
Regarding model selection:

Sorry, but while it is true that model selection is valuable and that there are many ways to do it, the debate about stepwise is pretty much over: It doesn't work. The output is wrong.

I favor using substantive knowledge.  The authors know more about the data and the models than any automatic algorithm does. But if they insist on using an algorithm, they should use one that attempts to adjust for the problems of stepwise.  I like LASSO but I would be fine with other penaized methods (there IS debate about which of these methods is best). 

They are correct that stepwise is used a lot. But ... that's not really a reason to keep using it.

We attempted to follow Reviewer 3’s suggestion to use LASSO instead of stepwise to do our automatic model building. To the best of our knowledge no R packages exist that could handle hierarchical models (this was important to us because we are interested in specific interactions). We did find a recently published package called ordinalNet ([https://cran.r-project.org/web/packages/ordinalNet/](https://urldefense.com/v3/__https:/cran.r-project.org/web/packages/ordinalNet/__;!!JYXjzlvb!wJJ__6b9HNYO_CF7ecj226MILHNrWAxMoeBkqPRzT8B5tZUR_fbrR9yzER7aNK4$)

). This is an elastic net, which hybridizes ridge and lasso regression. However, we are hesitant to proceed with it because, i) the package has not yet reached the level of maturity we want, but more importantly, ii) it is strictly speaking not LASSO and we are thus unsure whether this would satisfy Reviewer 3’s request. We are thus not optimistic that we can do automatic model building according to Reviewer 3’s specifications.

However, as reviewer 3 noted in the first round of revisions, they prefer that we *not* use automatic model building at all. They prefer a knowledge-driven approach. Our interpretation of this preference is that Reviewer 3 is concerned about the robustness of our statistical analysis. We have thus developed knowledge-based models and provided our results of these additional analysis as supplementary material. For convenience, we provide a brief description of what we did below.

Using our initial motivations for our study, we developed knowledge-based models, in which we investigate propensity to vaccinate as a function of: i) number of local cases (our surrogate for risk), ii) types of disease severity (morbidly and mortality), and iii) common socio-demographic predictors such as age, sex, education, income, religiosity, and ideology. We developed four models. The first looked at only main effects. The second looked at interactions between our risk variable (number of local cases) and all others. The third took into account interactions between our scenario types (morbidity and mortality) and all others. Finally, we considered a model with all pairwise interaction between the main effects and both our risk variable (number of local cases) and our scenario types (morbidity and mortality). Our results are extremely similar across these models and to our original model: number of cases and scenario type are significant (p<0.01), as well as age, sex, income, and political leaning. The one variable that our automatic approach (stepwise model selection) missed was related to commuting. The fact that our results are highly consistent across these different knowledge-based models (and our automatic model selection approach) suggest that our findings are robust across statistical approaches.
